# Polymorphism of the prolactin gene and its association with reproductive traits in F2 local crossed chickens

**DOI:** 10.14202/vetworld.2025.29-39

**Published:** 2025-01-09

**Authors:** Ega Rosalinda, Heru Sasongko, Dyah Maharani

**Affiliations:** 1 Department of Animal Breeding and Reproduction, Faculty of Animal Science, Universitas Gadjah Mada, Yogyakarta, Indonesia; 2 Department of Animal Production, Faculty of Animal Science, Universitas Gadjah Mada, Yogyakarta, Indonesia

**Keywords:** local crossed chickens, polymorphism, prolactin gene, reproductive traits, single-nucleotide polymorphisms

## Abstract

**Background and Aim:**

Reproductive traits are crucial for genetic improvement in chickens. Prolactin (PRL) is a gene involved in a complex hormonal network that regulates reproduction in chickens. In this study, three local chicken breeds were crossed and had been produce a second filial (F2) generation, referred to as the F2 local crossed chicken. This study aimed to evaluate reproductive traits, identify single-nucleotide polymorphisms (SNPs), and assess genetic variation in the PRL gene of F2 local crossed chickens.

**Materials and Methods:**

Data on reproductive traits, including body weight (BW) at first egg laying, total egg production (EP) at the first 90 days of laying eggs, egg weight (EW), egg length (EL), and egg width (EWd), and blood samples from 60 hens of F2 local crossed chicken (Merawang × Kampung Unggul Balitbangtan [KUB], n= 30; Murung Panggang × KUB [MP × KUB], n= 30) were collected. SNPs within PRL gene were identified using BioEdit version 7.0. Genetic diversity was calculated using Popgen 1.32. Statistical analysis of reproductive traits and its association with genotypes were assessed using Statistical Product and Service Solutions version 20.

**Results:**

Crossing patterns had no significant effect on reproductive traits (p > 0.05), except for EWd (p < 0.05). Five polymorphic SNPs were identified in exon 5 of the PRL gene: 8052T>C, 8113G>C, 8187C>T, 8188G>A, and 8321C>T. Observed and expected heterozygosity range from 0.15–0.52 and 0.14–0.38, respectively. All SNPs were in the Hardy-Weinberg equilibrium based on Chi-square test (χ^2^ <3.841), except for SNP 8052T>C in F2 crossing of MP × KUB. SNP 8052T>C was significantly associated with BW (p < 0.05), with TT genotype chickens showing higher BW. SNP 8187C>T was associated with EW and EL (p < 0.05), with CT genotype chickens having higher values for both traits.

**Conclusion:**

This study demonstrates the significant role of the PRL gene in influencing reproductive traits in F2 local crossed chickens. While crossing patterns showed limited impact, specific SNPs in exon 5 of the PRL gene were associated with BW, EW, and EL. The findings highlight PRL gene polymorphisms as valuable markers for improving reproductive traits in poultry breeding programs.

## INTRODUCTION

Chickens are essential livestock commodities, serving as a primary source of animal protein through egg and meat production. Indonesia possesses numerous local chicken genetic resources that are known for their excellent adaptability, high disease resistance, ease of maintenance, and low feed quality requirements [1]. Local chicken is also in high demand because of the savory taste of meat and relatively stable market prices [2]. However, the breeding of local chickens in Indonesia faces challenges such as low productivity, inefficient reproduction, the scarcity of superior breeds, and extended rearing periods [2]. This situation underscores the need to enhance the genetic quality of local Indonesian chicken through crossbreeding and selection. Crossbreeding is performed by combining both additive and non-additive genes from different livestock breeds to produce offspring with superior genetic traits compared to parents [3]. In this study, a crossbreeding program was developed to develop a new Indonesian chicken breed by crossing three different local chickens. Merawang and Murung Panggang chickens were used as male lines, whereas Kampung Unggul Balitbangtan (KUB) chicken was used as female line. This program produced a second filial (F2), which we will refer to as an F2 local crossed chicken. Merawang chicken is raised for both eggs and meat and originates from Merawang District in Bangka Belitung Province [4]. Murung Panggang chicken, from South Kalimantan Province, is mainly raised for meat and can reach 4 kg in 5 months [5]. The KUB chicken has been bred for over six generations and is known for its high egg production (EP) and low brooding behavior [6]. The evaluation of the reproductive profile of each chicken generation is crucial for enhancing genetic quality. In chicken breeding, selection often relies on economically valuable traits, including body weight (BW) at first egg laying, production quantity, weight, length, and width.

With advances in science, molecular selection has been proposed as an additional method for improving these traits. Molecular selection is a technique used to identify and select desirable genetic characteristics in livestock using DNA markers on specific genes that encode specific traits [7]. Therefore, integrating modern techniques with traditional knowledge can potentially enhance the productivity of Indonesian local chicken. Prolactin (PRL) is widely known as a candidate gene for selection in relation to reproductive traits. In chickens, the PRL gene is located on chromosome 2 and consists of four introns and five exons. It is a member of the transforming growth factor-β subfamily and plays an important role in physiological functions [8]. Furthermore, the gene encodes PRL hormone, which is produced by the anterior pituitary gland and plays various biological roles across vertebrates [9]. This hormone directly influences the hypothalamus-pituitary-gonadal axis, the main hormonal pathway controlling EP. In most cases, an increase in PRL levels leads to ovary regression and triggers incubation behavior [8, 9]. Polymorphisms in the PRL gene have been reported in various chicken breeds, including Qingyuan Partridge, Recessive White, White Leghorn, Yangshan, Taihe Silkies, White Rock, Nongdahe, Hubbard F15, Lohmann, Cobb 500, and Avian 48 chickens [9–11]. Moreover, studies have been conducted on various Indonesian local chicken breeds, including Papuan and IPB-D1 chickens [12, 13]. Previous studies by Li *et al*. [9], Mohamed *et al*. [11], and Rohmah *et al*. [13] identified single-nucleotide polymorphisms (SNPs) in the PRL gene that were significantly associated with various traits, including age at first egg laying, EP, and mortality rate.

Studies on PRL gene polymorphisms and their associations with reproductive traits in Indonesian local crossed chickens have not been conducted. Therefore, this study was conducted to provide preliminary information on the reproductive traits of locally crossed chickens and their associations with PRL gene polymorphisms. This study aimed to evaluate reproductive traits, identify SNPs, and assess genetic variations in PRL gene. The results will offer insights into significant DNA markers that can be used as selection tools for improving reproductive traits in the breeding of locally crossed chickens.

## MATERIALS AND METHODS

### Ethical approval

This study was approved by the Research Ethics Committee of the Faculty of Veterinary Medicine at Universitas Gadjah Mada, Yogyakarta, Indonesia (00016/ECFKH/Eks./2021).

### Study period and location

Chicken blood collection in this study was conducted in December 2021, and data collection for reproductive traits was conducted from April to June 2022 at Sambirejo Village, Semanu District, Gunungkidul Regency, Yogyakarta Special Region Province. Chicken DNA genome analysis was conducted from October 2023 to February 2024 at the Laboratory of Genetics and Animal Breeding, Faculty of Animal Science, Universitas Gadjah Mada.

### Schematic crossing patterns

**Figure 1:**
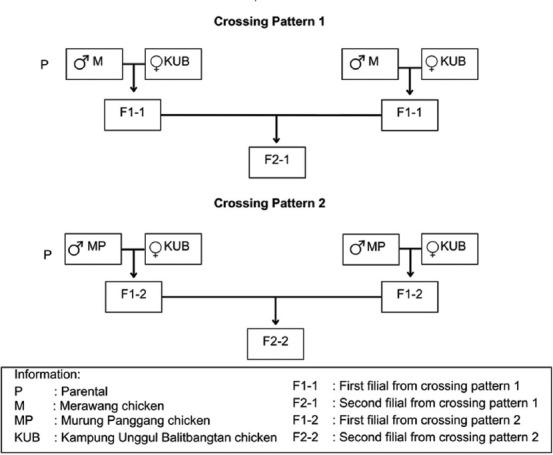
Scheme of crossing patterns of F2 local crossed chickens.

The F2 local crossed chickens in this study were obtained from crossing Merawang and Murung Panggang chickens as the male line, while KUB chickens represented the female line. Natural and inter-se mating methods were used, and the paired chickens were placed in a breeding cage with a ratio of roosters to hens of 1:5. A schematic of the crossing patterns used in this study is shown in Figure 1.

### Chicken management

A total of 60 chickens, consisting of each of 30 hens, of F2 Merawang × KUB (M × KUB) and F2 Murung Panggang × KUB (MP × KUB) were used. The chickens were intensively reared in a semi-closed house with an individual cage system and fed commercial layer feed, which has a composition of crude protein of 17% and metabolic energy of approximately 2,700 kcal/kg feed. Drinking water was provided *ad libitum* using a nipple drinker.

### Data collection for reproductive traits

Reproductive traits of F2 local crossed chickens for BW at first egg were obtained by measuring the weight after the chicken first laid egg. Total EP and egg weight (EW) were collected and weighed daily for 90 days. Egg length (EL) and egg width (EWd) were measured from samples collected at weeks 26, 29, and 32.

### DNA isolation, amplification, and sequencing

Approximately 0.5 mL of chicken blood was taken through the pectoralis vein using a 1 mL disposable syringe and inserted into a vacutainer containing ethylenediaminetetraacetic acid (EDTA). The samples were then stored in a freezer at a temperature of −25°C for further analysis. Chicken genomic DNA was isolated from whole blood using gSYNC^™^ DNA Extraction Kit cat. No. GS300 (Geneaid Biotech Ltd., Taiwan) according to the manufacturer’s instruction. The results for DNA isolation were confirmed with the electrophoresis method with 1% agarose gel (1^st^ Base, Malaysia) and 1× Tris-Boric-EDTA (TBE) buffer solution. The DNA bands were stained with Ethidium Bromide (EtBr). The DNA amplification was performed through the polymerase chain reaction (PCR) method using a Peqlab thermocycler machine (peqSTAR^®^, Peqlab, UK). A pair of primers, namely PRL-F: 5‘-CTG TTC TAC ACC CAG ACA GAT TGA-3’ and PRL-R: 5‘- AAG GTA TAA GCC ATC CCA GCT ATT-3’ [11], were used to amplify the 609-bp DNA fragment of the PRL gene based on GenBank accession no. AF288765.2. The sequence target consists of a partial area of intron 4 (59 bp), the entire area of exon 5 (418 bp), and a partial area of the terminator region (132 bp). A total of 25 μL of reaction volume were used for the amplification, consisting of 9.5 μL double distilled water, 12.5 μL PCR mix (2× MyTaq HS Red Mix, Bioline, USA), 0.5 μL each of forward and reverse primers, as well as 2 μL isolated chicken genomic DNA. Amplification was carried out for 35 cycles, starting with an initial denaturation for 5 min at 95°C, followed by denaturation for 30 s at 95°C, annealing for 30 s at 56°C, extension for 30 s at 72°C, and 10 min of final extension at 72°C. The amplification results were confirmed by electrophoresis using 1.5% agarose gel (1^st^ Base, Malaysia) and 1× TBE buffer solution. The DNA bands in the agarose gel were colored using EtBr. The PCR products were sent to the Laboratory of Integrated Testing and Research, Universitas Gadjah Mada, for sequencing using the Sanger Dideoxy method. The sequencing results are presented as electropherograms with different peak colors, where adenine (A), guanine (G), cytosine (C), and thymine (T) are represented by green, black, blue, and red, respectively.

### SNP identification and genotyping

The sequencing results for the targeted PRL sequence were edited, aligned, and genotyped using BioEdit version 7.0 software [14] for SNP identification. Each sequence was cut at the 5′ and 3’ ends, which were parallel to the attachment positions of the PRL-F and PRL-R primers. The edited sequences were then aligned, and GenBank accession no. AF288765.2 was used as a reference sequence to name the position of the SNP. Different nucleotides in the aligned sequences were categorized as SNPs. Genotyping was performed by checking the alignment results of the edited sequences on the electropherograms. A double-peak graph was considered a heterozygous genotype, whereas a single-peak graph corresponded to a homozygous genotype. Furthermore, amino acid change analysis was performed for the nucleotides located in the coding sequence using ExPasy [15]. The amino acid translation results were aligned to analyze changes using Clustal Omega [16].

### Statistical analysis

The effect of crossing patterns on reproductive traits was analyzed using an independent sample t-test in SPSS version 20 software [17], with a mathematical model as follows:


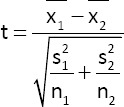


Where t is the t-test value; x_1_ is the mean of reproductive traits of F2 M × KUB; x_2_ is the mean of reproductive traits of F2 MP × KUB; s_1_ is the standard deviation of the reproductive traits of F2 M × KUB; s_2_ is the standard deviation of the reproductive traits of F2 MP × KUB; n_1_ is number of sample of the reproductive traits of F2 M × KUB; and n_2_ is number of sample of the reproductive traits of F2 MP × KUB. All data are presented as mean ± standard deviation.

Genetic diversity, including genotype and allele frequencies, as well as observed heterozygosity (Ho) and expected heterozygosity (He) to examine the PRL gene distribution in the population, and Chi-square test (χ^2^) for Hardy–Weinberg equilibrium were performed using Popgen 1.32 software [18]. The association between genotype in each SNP and reproductive traits was analyzed using the General Linear Model procedure in SPSS version 20 [17]. The mathematical model used was:


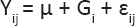


Here, Y is the reproductive traits of j^th^ individuals and i^th^ genotype; µ is the mean; G_i_ is the effect of i^th^ genotype on reproductive traits, and ɛ_ij_ is random error.

## RESULTS

### Reproductive traits of F2 local crossed chickens

This study used 60 F2 local crossed chickens, with 30 each in two crossing patterns: F2 M × KUB and F2 MP × KUB. The reproductive traits of two crossing patterns in F2 local crossed chickens are summarized in Table 1. Descriptively, the reproductive profile of F2 M × KUB was higher than that of F2 MP × KUB, except for EP. However, the results of the independent sample t-test showed that different crossing patterns had no significant effect (p > 0.05) on the reproductive traits of the chicken, except for EWd (p < 0.05). This indicated that different breeds did not affect the phenotypic traits of the chicken.

**Table 1:** Mean and standard deviation of reproductive traits of F2 local crossed chickens.

Variable	Crossing patterns				p-value

	n	F2 M × KUB	n	F2 MP × KUB	
BW (g)	30	2039.80 ± 311.54	30	1999.27 ± 211.88	0.558
EP 90	30	40.27 ± 12.75	30	41.47 ± 11.89	0.704
EW (g)	30	44.57 ± 3.61	30	43.79 ± 3.09	0.378
EL (mm)	30	50.92 ± 1.89	30	50.81 ± 1.58	0.821
EWd (mm)	30	39.59 ± 1.23^b^	30	38.92 ± 1.29^a^	0.045

BW=Body weight at first egg, EP 90=Total egg production at the first 90 days of laying eggs, EW=Egg weight, EL=Egg length, EWd=Egg width, n=Number of samples, F2 M × KUB=Second filial from crossing of Merawang chicken with KUB chicken, F2 MP × KUB=Second filial from crossing of Murung Panggang chicken with KUB chicken, ^a,b^=Different superscript in the same row indices significant difference between crossing patterns (p < 0.05)

### SNP identification of PRL

**Figure 2:**
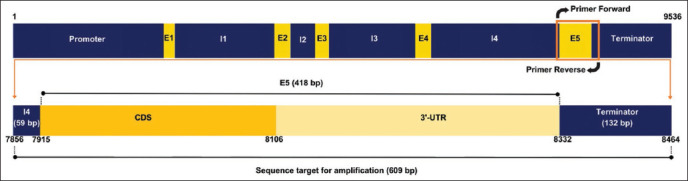
Illustration of sequence target for amplification of the PRL gene. The PRL gene has a total length of 9,536 bp based on GenBank accession no. AF288765.2. The sequence target is 609 bp, consisting of exon 5 and partial of intron 4 and terminator region. E1-E5= Exon 1-5, I1-I4= Intron 1-4, CDS=Coding sequence, 3′-UTR= 3′-untranslated region, bp=Base pairs.

**Figure 3:**
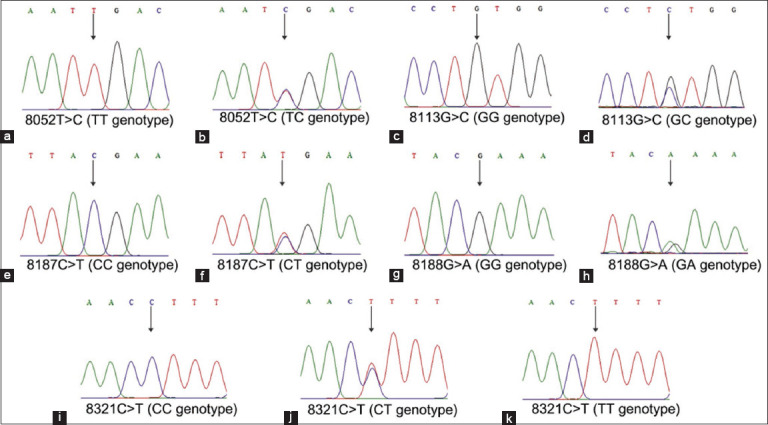
(a-k) Electropherogram of genotypes of single-nucleotide polymorphisms in exon 5 of the prolactin gene in F2 local crossed chickens.

Figure 2 illustrates the sequence target used in this study. A total of 609 bp fragments were successfully amplified and sequenced from the PRL gene of F2 local crossed chicken. A total of five SNPs were detected in exon 5 of the PRL gene, namely 8052T>C, 8113G>C, 8187C>T, 8188G>A, and 8321C>T (Table 2) following the position on GenBank with accession no. AF288765.2. All SNPs were invented for both crossing patterns. The SNPs 8052T>C and 8113G>C have been previously reported by Li *et al*. [9] and Rohmah *et al*. [13] in Qingyuan Partridge, Recessive White, and IPB-D1, whereas 8187C>T was found in a Hubbard F15 chicken [11]. The variation in SNP 8321C>T from F2 M × KUB was the only SNP that revealed three genotypes according to the electropherogram results (Figure 3). Furthermore, 8052C>T was the only SNP located in the coding sequence (Table 2). In this study, amino acid change analysis was only performed for SNPs located in the coding sequence, considering that this area would be translated into amino acids during the translation process. Based on the result, the different nucleotides of SNP 8052T>C (ATT>ATC/Isoleucine>Isoleucine) were categorized as silent mutations because they did not change the amino acid composition (Table 2).

**Table 2:** SNP position in the exon 5 of the PRL gene in F2 local crossed chickens.

SNP	Position	AA change	Mutation type
8052T>C	CDS	Iso/Iso	Silent
8113G>C	3’- UTR	-	-
8187C>T	3’- UTR	-	-
8188G>A	3’- UTR	-	-
8321C>T	3’- UTR	-	-

SNP=Single nucleotide polymorphism, CDS=Coding sequence, 3′-UTR=3′-untranslated region, AA=Amino acid, Iso=Isoleucine, PRL=Prolactin

### Genetic diversity of PRL

The genetic diversity of the PRL gene in the F2 local crossed chicken (Table 3) showed that the main homozygous genotypes, including TT for SNP 8052T>C, GG for SNP 8113G>C and 8188G>A, as well as CC for SNP 8187C>T and 8321C>T, were more prevalent than the heterozygous genotypes in both crossing patterns. However, an exception was observed for SNP 8052T>C in the F2 MP × KUB group, where the heterozygous genotype was more frequent than the homozygous. The allele frequencies varied from 0.02 to 0.98, with the highest frequency observed for the C allele at SNP 8187C>T. The heterozygosity results based on PRL gene distribution in the population showed that the observed heterozygosity (Ho) and expected heterozygosity (He) varied from 0.17 to 0.60 and from 0.15 to 0.42, respectively. All SNPs had higher Ho values than He, while the Chi-square test for the Hardy-Weinberg equilibrium indicated that all SNPs conformed to the Hardy–Weinberg equilibrium (χ^2^ <3.841), except for SNP 8052T>C in the F2 MP × KUB group, where the χ^2^ value exceeded the threshold of 3.841, indicating a deviation from equilibrium.

**Table 3:** Genotype and allele frequencies, heterozygosity, and Chi-square value (χ^2^) for Hardy-Weinberg equilibrium of the PRL gene from two crossing patterns in F2 local crossed chickens.

SNP	Crossing patterns	n	Genotype frequencies			Allele frequencies		H_O_	He	χ^2^-value
			TT	TC	CC	T	C			
8052T>C	F2 MXKUB	30	0.57	0.43	0.00	0.78	0.22	0.43	0.34	2.092^ns^
	F2 MPXKUB	30	0.40	0.60	0.00	0.70	0.30	0.60	0.42	5.153*
			GG	GC	CC	G	C			
8113G>C	F2 MXKUB	30	0.60	0.40	0.00	0.80	0.20	0.40	0.32	1.697^ns^
	F2 MPXKUB	30	0.53	0.47	0.00	0.77	0.23	0.47	0.36	2.549^ns^
			CC	CT	TT	C	T			
8187C>T	F2 MXKUB	30	0.73	0.27	0.00	0.87	0.13	0.27	0.23	0.612^ns^
	F2 MPXKUB	30	0.97	0.03	0.00	0.98	0.02	0.03	0.03	0.000^ns^
			GG	GA	AA	G	A			
8188G>A	F2 MXKUB	30	0.83	0.17	0.00	0.92	0.08	0.17	0.15	0.195^ns^
	F2 MPXKUB	30	0.57	0.43	0.00	0.78	0.22	0.43	0.34	2.093^ns^
			CC	CT	TT	C	T			
8321C>T	F2 MXKUB	30	0.53	0.40	0.07	0.73	0.27	0.40	0.39	0.001^ns^
	F2 MPXKUB	30	0.70	0.30	0.00	0.85	0.15	0.30	0.26	0.819^ns^

SNP=Single-Nucleotide Polymorphism, n=Number of samples, Ho=Observed heterozygosity, He=Expected heterozygosity, F2 M × KUB=Second filial from crossing of Merawang chicken with KUB chicken, F2 MP × KUB=Second filial from crossing of Murung Panggang chicken with KUB chicken, χ^2^_(0.05;1)_=3.841, ns=Non significant (χ^2^<3.841),

* =Significant (χ^2^>3.841), PRL=Prolactin

### Genotype-phenotype association

This study investigated the association between each SNP and reproductive traits in the F2 of local crossed chickens using a general linear model. Genotypes present in fewer than three individuals in each SNP were excluded from the association analysis to minimize bias in the results. Therefore, the TT genotype of SNP 8321C>T in F2 M × KUB and SNP 8187C>T in F2 of MP × KUB were not analyzed. According to the results presented in Tables 4 and 5, SNP 8052T>C in F2 MP × KUB was significantly associated with BW (p < 0.05). Chickens with the TT genotype had a higher mean of BW (2093.25 ± 202.35 g) than chickens with the TC genotype (1936.61 ± 199.24). Moreover, SNP 8187C>T in F2 crossing of M × KUB was significantly associated with reproductive traits, specifically EW and EL (p < 0.05). Chickens with the CT genotype had a higher mean for EW (47.28 ± 2.27 g) and EL (52.46 ± 1.22 mm) than those with the CC genotype, with mean of 43.63 ± 3.45 g and 50.36 ± 1.79 mm, respectively. There were no significant associations between SNP 8187C>T and BW, EP, and EWd (p > 0.05) in the F2 crossing of M × KUB. However, chickens with the CT genotype with the values 2110.88 ± 315.70 g, 43.50 ± 12.44, and 40.19 ± 0.78 mm, respectively for BW, EP, and EWd also showed a higher mean than chickens with the CC genotype with 2013.95 ± 313.35 g, 38.09 ± 12.58, and 39.37 ± 1.31 mm for the traits, descriptively.

**Table 4:** Association of SNPs in exon 5 of the PRL gene with BW, EP, EW, EL, and EWd in F2 M × KUB.

SNP	Phenotypes	n	Genotypes		p-value
			TT	TC	
8052T>C	BW (g)	30	2048.88 ± 325.19	2027.92 ± 305.43	0.687
	EP 90	30	36.00 ± 12.87	44.15 ± 10.95	0.078
	EW (g)	30	43.74 ± 3.71	45.73 ± 3.09	0.129
	EL (mm)	30	50.38 ± 2.00	51.61 ± 1.54	0.077
	EWd (mm)	30	39.41 ± 1.36	39.84 ± 1.06	0.353
			GG	GC	
8113G>C	BW (g)	30	2071.22 ± 329.41	1992.67 ± 290.07	0.809
	EP 90	30	36.94 ± 13.12	43.42 ± 11.10	0.171
	EW (g)	30	43.99 ± 3.76	45.52 ± 3.12	0.256
	EL (mm)	30	50.60 ± 2.14	51.40 ± 1.39	0.263
	EWd (mm)	30	39.49 ± 1.36	39.75 ± 1.05	0.588
			CC	CT	
8187C>T	BW (g)	30	2013.95 ± 313.35	2110.88 ± 315.70	0.169
	EP 90	30	38.09 ± 12.58	43.50 ± 12.44	0.305
	EW (g)	30	43.63 ± 3.45^a^	47.28 ± 2.27^b^	0.010
	EL (mm)	30	50.36 ± 1.79^a^	52.46 ± 1.22^b^	0.005
	EWd (mm)	30	39.37 ± 1.31	40.19 ± 0.78	0.111
			GG	GA	
8188G>A	BW (g)	30	2068.72 ± 316.92	1895.20 ± 263.94	0.281
	EP 90	30	38.40 ± 12.98	45.20 ± 9.31	0.277
	EW (g)	30	44.87 ± 3.68	43.25 ± 2.68	0.361
	EL (mm)	30	51.05 ± 2.02	50.26 ± 0.86	0.402
	EWd (mm)	30	39.67 ± 1.24	39.27 ± 1.27	0.531
			CC	CT	
8321C>T	BW (g)	28	2078.00 ± 368.29	1976.58 ± 249.30	0.697
	EP 90	28	38.69 ± 12.55	43.42 ± 11.52	0.317
	EW (g)	28	44.44 ± 3.74	45.22 ± 3.13	0.564
	EL (mm)	28	50.89 ± 2.04	51.09 ± 1.45	0.774
	EWd (mm)	28	39.59 ± 1.22	39.80 ± 1.23	0.652

SNP=Single-Nucleotide Polymorphism, BW=Body weight at first egg, EP 90=Total egg production at the first 90 days of laying eggs, EW=Egg weight, EL=Egg length, EWd=Egg width, n=Number of samples, F2 M × KUB=Second filial from crossing of Merawang chicken with KUB chicken ^a,b^=Different superscript in the same row indices significant difference between genotypes (p < 0.05), PRL=Prolactin

**Table 5:** Association of SNPs in exon 5 of the PRL gene with BW, EP, EW, EL, and EWd in F2 MP × KUB.

SNP	Phenotypes	n	Genotypes		p-value
			TT	TC	
8052T>C	BW (g)	30	2093.25 ± 202.35^b^	1936.61 ± 199.24^a^	0.045
	EP 90	30	41.42 ± 10.34	41.50 ± 12.64	0.985
	EW (g)	30	44.18 ± 4.40	43.54 ± 1.91	0.587
	EL (mm)	30	50.92 ± 1.62	50.72 ± 1.59	0.701
	EWd (mm)	30	39.08 ± 1.60	38.82 ± 1.08	0.603
			GG	GC	
8113G>C	BW (g)	30	2058.31 ± 203.46882	1931.79 ± 207.79619	0.104
	EP 90	30	40.81 ± 9.45	42.21 ± 13.97	0.747
	EW (g)	30	43.92 ± 3.95	43.65 ± 1.80	0.816
	EL (mm)	30	50.52 ± 1.66	51.15 ± 1.47	0.281
	EWd (mm)	30	38.96 ± 1.41	38.88 ± 1.19	0.870
			GG	GA	
8188G>A	BW (g)	30	2046.65 ± 202.79	1937.31 ± 215.21	0.165
	EP 90	30	40.12 ± 9.59	43.23 ± 13.99	0.476
	EW (g)	30	43.85 ± 3.841	43.72 ± 1.86	0.907
	EL (mm)	30	50.41 ± 1.67	51.35 ± 1.32	0.106
	EWd (mm)	30	38.90 ± 1.39	38.95 ± 1.22	0.935
			CC	CT	
8321C>T	BW (g)	30	1952.86 ± 211.25	2089.56 ± 197.43	0.109
	EP 90	30	41.57 ± 10.84	41.22 ± 13.88	0.941
	EW (g)	30	43.48 ± 3.22	44.50 ± 2.80	0.403
	EL (mm)	30	50.5376 ± 1.47	51.4578 ± 1.72	0.146
	EWd (mm)	30	38.9052 ± 1.18	38.9744 ± 1.61	0.896

SNP=Single-Nucleotide Polymorphism, BW=Body weight at first egg, EP 90=Total egg production at the first 90 days of laying eggs, EW=Egg weight, EL=Egg length, EWd=Egg width, n=Number of samples, F2 MP × KUB=Second filial from crossing of Murung Panggang chicken with KUB chicken ^a,b^=Different superscript in the same row indices significant difference between genotypes (p < 0.05), PRL=Prolactin

## DISCUSSION

### Reproductive traits of F2 local crossed chickens

The mean reproductive traits were higher in F2 M × KUB than in MP × KUB. This result is in contrast to the egg produced by the two crossing patterns, in which the mean of F2 MP × KUB was slightly higher than that of F2 M × KUB. Similar results were also reported in a previous study by Isaac and Obike [19] on Umudike local chicken, in which samples with heavier BW produced larger eggs but lower production than those with lower BW. The results can be attributed to the positive correlation between BW and EW. Chickens with high BW produce heavier eggs but have a low intensity of production, whereas smaller chickens lay more eggs, which are small [20]. The EP 90, especially for F2 M × KUB, was higher than that of a previous study by Alfiyanto *et al*. [21] on F1 M × KUB. An increase in EP can be caused by the selection effect. In general, selection is the process of selecting livestock with good performance for use as ancestors to produce the next generation that may have higher productivity [22].

The findings on the mean EW are consistent with the typical range for local chicken, which is 38–42 g in Kampung chicken [23]. In addition, the mean EL and EWd were consistent with those reported in previous study by Prawira *et al*. [24] on Kampung chicken. EW, EL, and EWd are crucial factors in chicken reproduction, with a positive correlation with hatching weight. An increase in EW generally indicates a larger egg size [25]. A larger egg size, including the EL and EWd, will influence the composition of the albumen and egg yolk. In general, larger eggs tend to have a higher internal composition as the initial size provides more nutrients and space for the developing embryo and leads to higher hatching weight [21].

Different crossing patterns showed no significant differences in reproductive traits (p > 0.05), except for EWd (p < 0.05) (Table 1). In this study, the same standards of BW were used as the selection criteria. Hence, the reproductive traits were similar. Selection significantly influences phenotypic appearance, leading to notable changes in physical traits over time. Carefully selecting animals with desirable characteristics enhances the traits within a population. Over time, this targeted selection resulted in a more uniform appearance of the animals [26].

### SNP identification of PRL

The PRL gene in chickens plays an important role in regulating reproductive traits through a complex hormonal pathway. PRL is synthesized and secreted by the anterior pituitary gland in response to various physiological signals, including light exposure and hormonal changes [27]. The hypothalamus tightly regulates this process through PRL-inhibiting factors, such as dopamine, and PRL-releasing factors, such as thyrotropin-releasing hormone [28]. After being released into the bloodstream, PRL binds to specific receptors on target cells in tissues such as the ovary, oviduct, and brain, triggering intracellular signaling pathways, including janus kinases-signal transducer and activator of transcription protein (JAK-STAT), which influence gene transcription associated with reproductive processes [29]. This suggests that genetic variations in PRL gene may modulate reproductive performance in chickens.

SNPs can be identified by analyzing variations in DNA fragments that are commonly present in an organism. SNPs are particularly useful for molecular markers, genetic mapping, and population diversity because of their high density and widespread distribution across the genome [30]. The SNPs in the PRL gene of F2 local crossed chickens were on exon 5. In previous studies by Li *et al*. [9] and Rohmah *et al*. [13] an SNP at 8052T>C was also found in Qingyuan Partridge, Recessive White, and IPB-D1 chicken. Another study of Qingyuan Partridge and Recessive White chicken identified an SNP at 8113G>C [9]. However, SNP 8187C>T has only been reported in Hubbard F15 chicken [11]. Exploration of the SNP located in exon 5 of the PRL gene suggests that this area has high polymorphism.

Based on the results, all SNPs found in F2-crossed local chickens were located in the exon region. An exon is a part of DNA that remains during mRNA maturation [31]. Mutations in exons can affect gene expression because this region includes a coding sequence, the area that is translated into amino acids. Then, the combination of amino acids is converted into a protein. However, several regions in the exon are non-protein-coding areas, including the 5′-cap, 5’-untranslated region (5’-UTR), 3’- untranslated region (3’-UTR), and poly-A tail, which are not translated into amino acids [32]. The SNP identification results showed that only SNP 8052T>C was located in the coding sequence, and the nucleotide change did not alter the amino acid composition. Previous studies by Li *et al*. [9] and Rohmah *et al*. [13] on the PRL gene in Qingyuan Partridge, Recessive White, and IPB-D1 chickens also reported a mutation in the same position. The condition in which variations in DNA-encoded sequences with the same amino acid composition occur is called a silent mutation [33].

### Genetic diversity of PRL

This study assessed genetic diversity by analyzing genotype and allele frequencies, heterozygosity, and Hardy–Weinberg equilibrium. Genotype frequency reflects the proportion or percentage of a specific genotype within a population [34]. In this study, we found that all SNPs only have two genotypes, with the exception of SNP 8321C>T in the F2 M × KUB having three genotypes. In previous studies by Li *et al*. [9] and Rohmah *et al*. [13] on Qingyuan Partridge, Recessive White, and IPB-D1 chicken, the CC genotype was present in SNPs 8052T>C and 8113G>C but had the lowest genotype frequency among the genotypes. In contrast, the CC genotype was absent in the present study, and its low frequency suggests a recessive nature for both SNPs. The unequal distribution of genotype frequencies for SNPs 8052T>C and 8113G>C in F2 local crossed chickens may be attributable to the selection program used in this study. This result is in line with the statement that a high frequency of homozygous genotypes in a population can lead to the absence of the recessive homozygous genotype and a low frequency of heterozygous genotypes, suggesting that selection may have influenced the population [35]. Moreover, all SNPs in this study had allele frequencies of <0.99. Similar results were observed in previous studies by Li *et al*. [9] and Rohmah *et al*. [13] using Qingyuan Partridge, Recessive White, and IPB-D1 chicken. An SNP is classified as polymorphic when its allele frequency is equal to or <0.99 [36].

By calculating genotype frequencies, heterozygosity can also be assessed in F2 local crossed chicken population. Heterozygosity indicates genetic variation because it reflects the proportion of heterozygous individuals within a population [37]. In this study, all Ho values were higher than He. A higher Ho value in the observed population may be due to crossbreeding activity. In principle, crossbreeding is the process of combining different genetic traits from two different breeds to produce a new genetic profile. The genetic combination occurs during meiosis in the process of cell division, where the possibility of crossing over occurs. Therefore, population diversity can increase due to the high possibility of heterosis caused by crossbreeding.

Based on the Chi-square test for the Hardy–Weinberg equilibrium, all SNPs are in agreement with the Hardy–Weinberg equilibrium except for SNP 8052T>C in F2 MP × KUB. The deviation indicates that selection and controlled mating in the population studied significantly impacted genotype frequencies, leading to an unbalanced genotype distribution. In contrast, SNPs at 8052T>C and 8113G>C in Qingyuan Partridge, Recessive White, and IPB-D1 chickens were in equilibrium [9, 13]. A SNP is considered to be in equilibrium when the Chi-square (χ^2^) value is less than the critical value in the Chi-square table. This study produced chickens by crossing two different breeds and managing mating practices. The selection was also carried out for each generation to select chicken with superior productivity. A population can be in equilibrium when genotype and allele frequencies remain stable from one generation to another, which is caused by random mating and no genetic change due to the large number of individuals in the population [38]. Several factors affect equilibrium in a population, including selection, mutation, migration, and controlled mating [39].

### Genotype-phenotype association

The association between each SNP in the PRL gene and reproductive traits was assessed to identify potential genetic markers for reproductive traits. Of the five SNPs discovered in this study, SNPs 8052T>C and 8187 C>T had significant effects on BW, EW, and EL (p < 0.05). Individuals with the TT genotype for SNP 8052 T>C in F2 MP × KUB had a higher BW value than individuals with the TC genotype, but this SNP did not affect the other reproductive traits. In contrast, a previous study by Rohmah *et al*. [13] reported that SNP 8052T>C affected EP at the first 90 days of laying eggs and did not have a relationship with BW in IPB-D1 chicken. Another study by Li *et al*. [9] on Qingyuan Partridge and Recessive White chicken also reported that SNP 8052T>C was significantly associated with total EP in the 300 days of age and age at first laying egg. Li *et al*. [9] also reported that SNP 8113G>C had a significant effect on total EP in the 300 days of age and age at first laying egg in Qingyuan Partridge and Recessive White chicken. In contrast, this SNP was not significantly associated with any reproductive traits. The variation in SNP 8187 C>T was also significantly associated with EW and EL, specifically in F2 M × KUB. Chickens with the CT genotype had higher EW and EL values than those with the CC genotype. Another study reported that SNP 8187 C>T had a significant relationship with total EP and mortality rates in Hubbard F15, Lohmann, Cobb 500, and Avian 48 chickens [11].

Given the significant association of SNP 8052T>C with BW in F2 MP × KUB (p < 0.05), a silent mutation can still affect the function of PRL, although it does not change the amino acid sequence. Silent mutations can alter mRNA translation speed, thereby affecting protein production efficiency [13]. A silent mutation in PRL could influence egg-laying behavior or growth rates by affecting PRL levels, even when the protein remains structurally unchanged [27]. Moreover, a significant association of SNP 8187C>T with EW and EL in F2 M × KUB (p < 0.05) indicated that the mutation affected the function of the PRL gene, despite being located in the 3′-UTR. The 3′-UTR plays a crucial role in the regulation of gene expression. This region is located downstream of the coding sequence and upstream of the polyadenylation site. The 3′-UTR also plays a crucial role in multiple post-transcriptional regulatory processes because it controls mRNA stability, translation efficiency, and mRNA localization [40]. One of the primary functions of mRNA stability is to determine whether certain sequences within the 3′-UTR can either stabilize the mRNA or serve as targets for degradation. In addition, the 3′-UTR contains binding sites for microRNAs and RNA-binding proteins, which can repress or enhance translation by affecting the binding of ribosomes. This region is also implicated in the subcellular localization of mRNA, directing it to specific cellular compartments, which are essential for localized protein synthesis [41]. The 3′-UTR contributes to fine-tuning gene expression through these mechanisms, ensuring that proteins are produced at the right time, place, and quantity within the cell.

## CONCLUSION

This study identifies significant genetic polymorphisms in the PRL gene and their associations with reproductive traits in F2 local crossed chickens. The findings demonstrate that crossing patterns generally have a limited impact on reproductive profiles, except for egg width. However, specific SNPs, particularly 8052T>C and 8187C>T, are strongly associated with traits such as body weight, egg weight, and egg length. These SNPs exhibit significant potential as genetic markers for selective breeding programs. The study highlights the importance of leveraging genetic diversity and molecular tools to enhance poultry productivity.

Future research should validate these findings across larger populations and explore the functional roles of identified SNPs to gain deeper insights into their genetic mechanisms. Practical applications of this research include the integration of SNP markers into marker-assisted selection frameworks to improve economically significant traits in poultry. Such advancements promise to enhance genetic improvement strategies, increase productivity, and contribute to sustainable poultry farming practices.

## AUTHORS’ CONTRIBUTIONS

DM and HS: Designed the study and revised the manuscript. ER: Collected the samples, conducted the study, analyzed the data, and drafted the manuscript. All authors have read and approved the final manuscript.
